# Rush to judgment: the STI-treatment trials and HIV in sub-Saharan Africa

**DOI:** 10.7448/IAS.18.1.19844

**Published:** 2015-05-18

**Authors:** Eileen Stillwaggon, Larry Sawers

**Affiliations:** 1 Department of Economics, Gettysburg College, Gettysburg, PA, USA;; 2 Department of Economics, American University, Washington, DC, USA

**Keywords:** HIV, AIDS, RCT, STI, sub-Saharan Africa, sexually transmitted disease and HIV, randomized controlled trial

## Abstract

**Introduction:**

The extraordinarily high incidence of HIV in sub-Saharan Africa led to the search for cofactor infections that could explain the high rates of transmission in the region. Genital inflammation and lesions caused by sexually transmitted infections (STIs) were a probable mechanism, and numerous observational studies indicated several STI cofactors. Nine out of the ten randomized controlled trials (RCTs), however, failed to demonstrate that treating STIs could lower HIV incidence. We evaluate all 10 trials to determine if their design permits the conclusion, widely believed, that STI treatment is ineffective in reducing HIV incidence.

**Discussion:**

Examination of the trials reveals critical methodological problems sufficient to account for statistically insignificant outcomes in nine of the ten trials. Shortcomings of the trials include weak exposure contrast, confounding, non-differential misclassification, contamination and effect modification, all of which consistently bias the results toward the null. In any future STI-HIV trial, ethical considerations will again require weak exposure contrast. The complexity posed by HIV transmission in the genital microbial environment means that any future STI-HIV trial will face confounding, non-differential misclassification and effect modification. As a result, it is unlikely that additional trials would be able to answer the question of whether STI control reduces HIV incidence.

**Conclusions:**

Shortcomings in published RCTs render invalid the conclusion that treating STIs and other cofactor infections is ineffective in HIV prevention. Meta-analyses of observational studies conclude that STIs can raise HIV transmission efficiency two- to fourfold. Health policy is always implemented under uncertainty. Given the known benefits of STI control, the irreparable harm from not treating STIs and the likely decline in HIV incidence resulting from STI control, it is appropriate to expand STI control programmes and to use funds earmarked for HIV prevention to finance those programmes.

## Introduction

Genital inflammation, lesions and HIV shedding caused by some sexually transmitted infections (STIs) are thought to promote HIV transmission and acquisition. Numerous observational studies in sub-Saharan Africa provide corroboration [1–14]. In the early 1990s, the first randomized controlled trial (RCT) to test the hypothesis that improving treatment of STIs reduces HIV incidence was conducted in Mwanza, Tanzania [15]. It found HIV incidence in the treatment arm 38% lower than among controls, but nine subsequent trials examining the hypothesis did not find statistically significant results [16–24].

A number of articles offer explanations for why the post-Mwanza STI-HIV trials did not replicate Mwanza's success. One explanation is that STI control is less likely to affect HIV incidence in mature epidemics [11,25–31] or where STI prevalence is low [12,27,32]. Moreover, reductions in risky sexual behaviours among controls [24,29,33] and enhanced prevention services to controls [11,29,33] likely eroded differences in HIV incidence between the trials’ arms. Most trials were underpowered as a result of lower than anticipated HIV incidence among participants [29]. Some trials might have had adherence problems or incorrect dosages of tested medication [11,29,32]. Most trials found few significant differences in STI incidence or prevalence outcomes [32]; only two trials found statistically significant differences for more than two STI outcomes [19,21]. All but one of the trials did not fully capture the effect of STIs on HIV transmission and instead measured only the effect of STIs on HIV acquisition [34]. Most trials addressing bacterial STIs could have been confounded by herpes simplex virus, HSV-2 [17,25,27,32,35]. Finally, in one community-based trial, a substantial share of incident HIV infections came from partnerships with people outside the community, whose treatment status was unknown and were likely untreated; the resulting exposure misclassification could have obscured the effects of interventions on HIV incidence within the study communities [36] (see also [37]).

Some commentators maintain that the post-Mwanza trials cast considerable doubt on the proposition that STI-control programmes can reduce HIV incidence. Prominent examples include Gray and Wawer [32] and Larson, Bertozzi and Piot [38], who conclude that funds earmarked for HIV prevention should not be spent on STI control because it is not yet certain that controlling STIs helps to prevent HIV. In contrast, others downplay the post-Mwanza trials because of their numerous shortcomings and argue that observational studies already provide sufficient evidence to justify STI control as HIV-prevention programming [11] (see also [29]).

This article analyses the 10 trials that examined the impact of STI control on HIV incidence in sub-Saharan Africa discussed in previous reviews [11,29,31,32,39]. Our argument builds on those reviews and other commentary [11,25,26–35,39–42] but presents a more detailed evaluation of the design of the 10 STI-HIV trials than earlier works, drawing upon a substantial literature on the methodology of RCTs [43–54].

## Discussion

We find that both design and implementation deficiencies led to weak exposure contrast, confounding, non-differential misclassification bias, contamination and effect modification in the post-Mwanza STI-HIV trials.

### Weak exposure contrast

An RCT tests whether differences between interventions (also called exposures) in treatment and control arms produce different outcomes. All of the post-Mwanza trials included important interventions designed to reduce HIV incidence that were identical in both arms. There were other interventions that differed between treatment and control arms, but all of the post-Mwanza trials delivered treatment and/or other HIV-prevention services to control arms that led to weak exposure contrast between arms, which made it difficult to detect statistically significant differences in HIV incidence between arms.

Before the Mwanza trial tested the hypothesis that improved treatment of STIs could reduce HIV incidence, there was the assumption of equipoise. The control communities in Mwanza received no interventions until after the trial was completed. Mwanza's success, however, changed the ethical calculus in the design of STI-HIV trials by providing evidence that STI-control programmes could affect HIV incidence. Given the serious harm from untreated STIs and the apparent impact on HIV incidence, ethics required that future trial designs incorporate treatment of identified STIs plus other interventions to reduce risk for all participants. Thus, the weak exposure contrast in the post-Mwanza trials was dictated by ethical considerations.

Because the ability of trials to find significant results depends on differences in interventions, we organize our discussion of the trials according to the interventions tested (i.e. those differing between treatment and control arms). Previous reviews [11,29,31] categorized the trials by level of randomization (assignment of individuals vs. communities to treatment or control arms) and whether trials tested bacterial or viral STIs. Those approaches take the focus away from the interventions and thus away from the weak exposure contrast, which in our view is the central problem of the post-Mwanza trials. As noted above, some commentary on the trials mentions weak exposure contrast (without using the phrase) as one of many problems, but none thoroughly explores the issue. (Table 1 provides detail about the trials’ interventions and explains our names for them.)

**Table 1:** Description of STI-HIV trials

Name of trial^a^ Location(s) *Lead author*, date Population	Treatment-arm interventions	Control-arm interventions
***Trials in which the tested interventions were applied to communities***		
**Mwanza** Mwanza, Tanzania ***Grosskurth*** [15], 1995 Six matched pairs of communities randomly assigned to treatment or control	–Efforts to improve delivery of STI treatment services, including establishment of STI reference clinic, training health centre staff, supervisory visits to clinics, stocking clinics with STI drugs and providing information on STIs to the community	
	***Interventions in both arms*** –Baseline and follow up interviews and examinations in sample (*N* ~ 1000) in each community –Everyone interviewed treated by clinician for symptomatic bacterial STIs and other illnesses –STI treatment in existing primary health care clinics	
**Masaka** Masaka, Uganda ***Kamali*** [16], 2003 18 rural communities randomly assigned to Treatment Arm A, Treatment Arm B or control arm	–A and B communities: information, education and communication activities –B communities: efforts to improve STI treatment by training health care workers in syndromic management of STIs and stocking clinics with STI drugs	–Community development activities (such as technical help and supervision for self-support and for-profit groups) and general health-related activities (such as home-based care for the elderly and health promotion seminars)
	***Interventions in both arms*** –Social marketing of condoms and voluntary counselling and testing for HIV	
**Zimbabwe-East** Manicaland, Zimbabwe ***Gregson*** [17], 2007 Six matched pairs of communities randomly assigned to treatment or control	–Peer education and condom distribution among female sex workers and male clients funded by microfinance projects –Programmes to strengthen STI treatment in government clinics, including stocking STI medications –Open days at health centres with activities to promote behaviour change	
	***Interventions in both arms*** –Distribution and social marketing of condoms –AIDS awareness meetings, posters and leaflets –Syndromic STI treatment in government clinics	
***Trials in which the tested interventions were applied to individuals or couples***		
**Rakai** Rakai, Uganda ***Wawer*** [18], 1999 All consenting residents aged 15 to 59 in 10 community clusters, which were randomly assigned to treatment or control and visited every 10 months	–Presumptive treatment with azithromycin, ciprofloxacin and metronidazole –Intramuscular penicillin for serologically identified syphilis; treatment in the home within 24 hours of diagnosis	–Those reporting STI symptoms referred for free treatment in mobile clinics providing general health care in the village at the time of the visit –Presumptive treatment with antihelminthics and nutritional supplements –Those with positive syphilis serology referred to government clinics for free treatment
	***Interventions in both arms*** –Community education programmes for HIV prevention, counselling, free condoms and free health care at mobile clinics present in village at time of household visits –Those with STI symptoms between visits advised to seek treatment at government clinics	
**Rakai-Maternal** Rakai, Uganda ***Gray*** [19], 2001 All consenting pregnant women in 10 community clusters, which were randomly assigned to treatment or control and visited every 10 months (nested in the Rakai trial [18])	–Presumptive treatment with azithromycin, cefixime and metronidazole by project staff –Intramuscular penicillin for serologically identified syphilis by project staff	–Symptomatic bacterial STIs treated syndromically at time of survey by project staff –Those with positive syphilis serology referred to government clinics for free treatment –Presumptive administration of nutritional supplements
	***Interventions in both arms*** –Health education, condom promotion, HIV-prevention counselling and free health care at mobile clinics –Those with STI symptoms between visits advised to seek treatment at government clinics	
**Abidjan** Abidjan, Côte d'Ivoire ***Ghys*** [20], 2001 Female sex workers (*N*=542) attending STI screening facility randomly assigned to treatment or control	–Monthly clinic visits with examination, testing for STIs and syndromic treatment	–Monthly clinic visits with examination for STIs if reporting vaginal discharge, abdominal pain or genital ulcer
	***Interventions in both arms*** –Every six months, examination and testing for STIs and HIV and treatment for all diagnosed bacterial STIs –Monthly health education and free condoms	
**Nairobi** Nairobi, Kenya ***Kaul*** [21], 2004 Female sex workers (*N*=466) randomly assigned to treatment or control	–Monthly doses of azithromycin	–Monthly placebos
	***Interventions in both arms*** –Prompt treatment of any symptomatic bacterial STI at monthly visit –Screening and therapy for asymptomatic bacterial STIs every six months –HIV and STI prevention counselling and free condoms	
**NW-Tanzania** Mwanza, Shinyanga and Tabora ***Watson-Jones*** [22], 2008 Female food and recreational facility workers (*N*=821) with HSV-2, randomly assigned to treatment or control	–Daily doses of acyclovir	–Daily placebos
	***Interventions in both arms*** –Blood testing every three months –Vaginal specimens taken at 6, 12, 24 and 30 months –Examination at 3, 9, 15, 21 and 27 months if symptomatic –Syndromic management of any symptomatic STIs and treatment of laboratory-confirmed STIs –STI- and HIV-prevention counselling, free treatment for minor medical conditions and free condoms	
**3-Cities**^b^ Johannesburg, Harare and Lusaka ***Celum*** [23], 2008 HSV-2 seropositive women (*N*=1358) randomly assigned to treatment or control	–Daily doses of acyclovir	–Daily placebos
	***Interventions in both arms*** –Treatment of HSV-2 ulceration diagnosed at monthly visits with five-day course of acyclovir –Monthly visits (and examinations if STI symptoms reported) plus quarterly examinations for diagnosis and treatment of STIs and HSV-2 ulceration –STI- and HIV-prevention counselling –Free condoms	
**14-Cities** Fourteen cities in sub-Saharan Africa ***Celum*** [24], 2010 Discordant couples (*N*=3408) in which the HIV-positive partner was also HSV-2 positive randomly assigned to treatment or control	–Daily doses of acyclovir	–Daily placebos
	***Interventions in both arms*** –Those who were diagnosed with HSV-2 in quarterly examinations and/or who reported symptoms consistent with HSV-2 ulceration in monthly interviews were treated with five-day course of acyclovir –Quarterly examinations for diagnosis and treatment of STIs –STI- and HIV-prevention counselling –Referral for HIV-positive partners to clinics offering ART –Free condoms	

a In the literature, the trials are referred to in a variety of ways, according to the lead author, the site of the trial or the interventions. We use a uniform, geographic designation to identify the trials.

b Trial included men who have sex with men in Peru and the United States, who fall outside the purview of this article.

STI, sexually transmitted infection.

#### Seven trials testing differences in medication or examination regimens

In the Nairobi trial, participants in the treatment group were given antibiotics presumptively each month. Controls visited a clinic monthly and were treated for symptomatic bacterial STIs. Moreover, every six months, all participants were screened, examined and treated for symptomatic and asymptomatic bacterial STIs [21].

In Abidjan, treatment group participants were examined for STIs monthly. Controls reporting STI symptoms at monthly visits were examined. In both arms, bacterial STIs diagnosed by examination were promptly treated [20]. Every six months, laboratory tests were used to screen all participants for STIs, and all diagnosed bacterial STIs were treated.

Rakai [18] tested different ways of administering antibiotics for STIs every 10 months: mass drug administration (MDA) in the treatment-arm communities versus referring symptomatic controls for free treatment in mobile clinics present in the community at the time of testing (and to government clinics if positive for syphilis). MDA is sometimes understood as a community-level intervention because its objective is to reduce population prevalence. The community protection occurs, however, because individuals with the target infection are treated, not because uninfected individuals are treated. Although randomization in the trial was at the community level and there were identical community-level interventions in the two arms (for example, free condom distribution at various sites in the community), the interventions that differed between arms – presumptive medication or referral for treatment – were applied to individuals. Those interventions differed between arms to the extent that individuals in the control arm were non-compliant or asymptomatic. Because many STIs are asymptomatic and probably not everyone sought the treatment to which they were referred, the trial's interventions had different effects on STI prevalence and incidence in treatment and control communities. Nevertheless, in Rakai as in all of the post-Mwanza trials, controls received important interventions that weakened exposure contrast and help explain the inability to find statistically significant results.

Rakai-Maternal [19] project staff tested all participants for syphilis, medicating positive treatment-arm participants and referring positive controls to government clinics. Project staff administered antibiotics to all treatment-arm participants and to all controls with symptomatic bacterial STIs other than syphilis. Interventions differed between arms because asymptomatic bacterial STIs other than syphilis were not treated among controls (assuming individuals referred for syphilis treatment complied).

All three acyclovir trials (NW-Tanzania, 3-City and 14-City) tested the difference between presumptive treatment with daily doses of acyclovir to suppress HSV-2 ulceration versus treatment with acyclovir if diagnosed with HSV-2 ulcers at monthly [23,24] or quarterly [22] examinations. The 3-City trial reported that 31% of controls were treated with acyclovir during the trial [23]. All participants in both arms were treated for bacterial STIs using the same diagnostic procedures and medications.

The results of the seven STI-HIV trials with individual-level interventions can be summarized as follows: the trials demonstrate that presumptive STI treatment for the treatment arm provides about the same level of protection against HIV transmission as the state-of-the-art treatment of symptomatic STIs provided to the control arm when combined with established HIV-prevention services provided to all trial participants.

None of the seven trials provides any evidence that treating STIs, whether presumptively or after diagnosis, is ineffective in reducing HIV incidence. Nevertheless, most reports of the trials and additional commentary written by authors of the trials summarize the results with assertions to that effect, without reference to the numerous interventions in the control arms. One review of the trials writes that they “demonstrated unequivocally that herpes suppressive therapy using a currently available treatment regimen was ineffective in reducing the acquisition or transmission of HIV” [11]. One trial asserts that “daily acyclovir therapy did not reduce the risk of transmission of HIV-1” [23]. Another says, “We observed no effect of the STD intervention on the incidence of HIV-1 infection” [18]. Again, “the results of this trial indicate that … acyclovir at a dose of 400 mg twice daily is not a viable public health intervention” [22]. Finally, “our results show that suppressive therapy with standard doses of acyclovir is not effective in reduction of HIV-1 acquisition” [23].

By taking the focus away from exposure contrast (comparisons between arms), these statements give the incorrect impression that the trials provide evidence that interventions to reduce STIs have no effect on HIV incidence. The trials, however, could only show the difference in outcomes between arms; the interventions in both treatment and control arms could have been effective in reducing HIV incidence or not. The lack of statistically significant differences in outcomes between arms cannot be construed as evidence about the effect of STI treatment on HIV incidence in either arm.

#### Three trials testing differences in community-level interventions

The Mwanza trial implemented community-level interventions to strengthen syndromic treatment of STIs in treatment communities but not in control communities. Mwanza was the only trial with substantial exposure contrast and significant results.

The Zimbabwe-East trial had many important HIV-prevention interventions that were identical in the two arms. Interventions designed exclusively for the treatment arm were as follows: (1) microfinance projects to fund peer education and condom distribution, (2) programmes to strengthen STI treatment in health centres, and (3) open days at health centres with activities to promote behaviour change. Deterioration of the Zimbabwean economy undermined the effectiveness of all three interventions, thus weakening exposure contrast. Economic problems caused the following: (1) cancellation of the microfinance programme, (2) shortages of fuel and medicine that undermined the programme to strengthen STI treatment in health centres and (3) fewer than planned health centre open days [17]. Exposure contrast was further diminished by unrelated organizations that sponsored peer-education programmes and other HIV-prevention activities in control communities. Moreover, in treatment communities, messaging by other agencies conflicted with the messaging promoted by the trial's interventions [17].

In Masaka, Quigley *et al*. found that some participants attended meetings in neighbouring communities assigned to a different arm [42]. That contamination reduced the trial's actual (in contrast to designed) exposure contrast. Neither Quigley *et al*. nor Kamali *et al*. [16] report using statistical methods that correct estimates of the trial's results for contamination.

Economic chaos in Zimbabwe-East and contamination in Masaka weakened actual exposure contrast, but both trials found that some interventions produced significantly lower HIV incidence. In Zimbabwe-East, there were AIDS awareness meetings in both arms. Among men who attended the meetings, HIV incidence and reported unprotected sexual encounters were 52 and 55% lower than among men who did not (both significant at the 0.05 level) [17]. In Masaka, HIV incidence among men (most of whom were in the treatment arm but some of whom were controls) who attended informational meetings addressing HIV and STIs was 68% lower than among those who did not (significant at the 0.045 level); for women attending the meetings, HIV incidence was 65% lower (significant at the 0.01 level) [42].

### Confounding

It has been often noted that the high prevalence of HSV-2 may have confounded the results of all the trials treating only bacterial STIs [17,18,21,25,27,35]. Identifying confounders depends on how the exposure is defined since a confounder must be associated with both the exposure and the outcome. If the exposure in the bacterial STI trials is just one or several bacterial STIs, then HSV-2 is not a confounder unless one posits that HSV-2 piggybacks on the other STIs (and is thus related both to exposure and outcome). Alternatively, if the exposure is the treatment intervention itself, that would also mean HSV-2 cannot be a confounder in the bacterial STI trials. More commonly, however, authors discussing the trials treat sexual behaviour as the exposure [1,8,10,21,25,27,32] because HIV and STIs share a mode of transmission, thus confounding the effects [25,32]. If sexual behaviour is considered the exposure, confounding is more serious in the STI-HIV trials than is generally understood. The trials and discussions of confounding in the trials overlook viral STIs other than HSV-2 that could have cofactor effects on HIV transmission, for example human papilloma virus [55]. More generally, just as any viral STI would confound the results of any bacterial trial, any bacterial STI could confound the results of the HSV-2 trials under the broader definition of exposure.

However the exposure is defined, what is important for the trials is that several different STIs (both viral and bacterial) are common in the population and none of the trials measured the effect of treating both kinds. Both viral and bacterial STIs are associated with higher risk of HIV. Even if treating one kind of STI has a beneficial effect in reducing risk of HIV acquisition or transmission, that effect is less likely to be observable (statistically significant) because of the effect of all the other STIs.

The STI-HIV trials pose another problem. Confounding should be eliminated or reduced in an experiment (such as an RCT) through randomization. The larger the number of random assignments in an experiment, the more closely the distribution of known and unknown risk factors in randomly assigned arms will resemble each other, reducing bias from confounding [48]. What matters, however, is not the number of participants in the trial but the number of randomizations [48, p143; 54, p993]. Half of the trials – Mwanza, Rakai, Rakai-Maternal, Zimbabwe-East and Masaka – had between 5 and 12 random assignments. With so few randomizations, one cannot rule out substantial confounding. Furthermore, it is likely that multiple confounders acted in the same direction to promote HIV transmission and bias trial results toward the null.

Another way to reduce confounding is restriction of study subjects. Restricting participants to persons with just one STI, however, would have entailed costly testing and continued surveillance beyond the means of the trials’ authors. Restriction, moreover, cannot control for unknown (or unrecognized) confounders [48, p142] and so imposes high information costs on trials.

### Microbial communities and genital health

Genital morbidities that are not sexually transmitted can also affect HIV outcomes. Symptoms of STIs (ulceration, inflammation, HIV shedding) that are considered mechanisms that increase HIV transmission efficiency are also symptoms of non-sexually-transmitted parasitic, bacterial and fungal genital infections that are highly endemic in all trial sites. Because they are not related to the exposure that causes STIs (sexual contact), they are not confounders. Nonetheless, they are important because the complexity of the genital microbial community generates substantial statistical noise in a trial that seeks to test the effect of treating just one or a few genital infections. Two of the ten trials reported that only about half of genital ulcers were of STI origin: 49% in Rakai [18] and 53% in Masaka [56]. Others have mentioned – but without elaboration – that non-sexually-transmitted genital morbidity could have reduced the post-Mwanza trials’ ability to find significant results [32].

Approximately 125 million people in sub-Saharan Africa are infected with *Schistosoma haematobium* [57], which is far higher than estimates of bacterial STI prevalence in the region (Table 1 in [58]) and about the same as estimates of HSV-2 (Tables 2 and 3 in [59])*. S. haematobium* larvae are transmitted through skin contact with contaminated water [60–62]. Sequelae include genital ulceration, inflammation, increased HIV shedding and other serious morbidities. The STI trials were all conducted in countries with a high burden of schistosomiasis [63]. In one study, 60% of women with *S. haematobium* infection had genital manifestations [64]. Four other studies reported 30 to 75% of women in endemic areas had reproductive tract infections of schistosomiasis, with infestation of worms and ova in the vagina, uterus, vulva or cervix [65]. In a community in rural Zimbabwe, 46% of women had genital lesions caused by schistosomiasis [66,67]. Only a single STI-HIV trial [18] even mentions schistosomiasis (but without distinguishing between *S. haematobium* and *Schistosoma mansoni*), and none administered the antischistosomal drug, praziquantel.

Schistosomal ulceration has been associated with three- and fourfold higher HIV prevalence in two studies [66,68]. The magnitude of that effect is comparable to the effect of STIs on HIV; three meta-analyses of observational studies find that STIs raise the risk of HIV transmission between two- and fourfold [11,13,14]. Given the high prevalence in sub-Saharan Africa of *S. haematobium* infection, its population attributable fraction for HIV in the region is likely to be substantial.

Some genital ulcers caused by abrasions become infected with *Streptococcus* or *Staphylococcus* bacteria [56]. Ulcers initially caused by bacterial or viral STIs or schistosomiasis also can become superinfected with Strep or Staph, preventing healing despite treatment with antibiotics, acyclovir or praziquantel. In Masaka, 18% of ulcers examined were positive for Staph or Strep [56]. No other trial mentions such ulcers and only two report using broad-spectrum antibiotics effective against those bacteria [18,19].

Bacterial vaginosis is not generally transmitted sexually, cannot be treated with the antibiotics typically used for STIs and can be recurrent. Bacterial vaginosis has been shown to increase risk of HIV acquisition by disrupting genital microbial communities [69–75]. Several of the STI-HIV trials reported substantial prevalence of bacterial vaginosis among participants in both arms [18,19,21–23] or in the district in which the trial was conducted [15], but only two reported post-trial prevalence of bacterial vaginosis significantly lower in the treatment arm than among controls [18,19].

Genital fungal infections can also produce inflammation and ulceration. The most common is candidiasis, which can double women's risk of acquiring HIV [10,76]. Only three trials even mention fungal infections [18,20,21].

There are two consequences of the failure of the 10 trials to target all genital infections that could enhance efficiency of HIV transmission. First, it constitutes non-differential misclassification (simply put, omitting important explanatory variables), which can bias results toward the null. Second, biological interaction between genital infections could result in effect modification, potentially invalidating the statistical tests used by the trials. The scope and direction of the distortion depend on the nature of the biological interaction, the statistical model tested and the choice of outcome measures [48, p202–8]. Restriction of study subjects to those without other genital morbidities could have improved the accuracy of testing STI interventions, but again, it would have entailed a time-consuming and costly search for eligible subjects. In sum, the failure of the trials to account for the complex genital environment in which sexual transmission of HIV occurs further undermines confidence in the trials’ results [77].

## Conclusions

The STI-HIV trials failed to show that efforts to control symptomatic STIs are consistently effective in reducing HIV incidence. That failure is not surprising given the weak exposure contrast, confounding, non-differential misclassification, contamination and effect modification, all of which biased the trials’ results consistently toward the null. The problem with the trials was primarily their design, not their implementation.

There are two important reasons why additional STI-HIV trials would have the same design flaws as the previous trials. First, ethical considerations will continue to necessitate minimal exposure contrast. Second, the purpose of an RCT is to test the impact of a single factor. RCTs are especially suited to tests such as drug trials when efficacy and side effects are unknown. They are not generally suited to public health interventions [52] where multiple diseases interact and recovery exhibits hysteresis, including schistosomiasis lesions in adult women and superinfections that are refractory to treatment. There are multiple benefits from treating STIs and other infections in the genital microbial community, but an RCT is unlikely to identify statistically significant results due to confounding, non-differential misclassification and effect modification. Thus, we do not advocate more STI-HIV trials.

Before the STI-HIV trials were launched, STI treatment was known to be efficacious (for STIs), with minimal side effects. The results of the Mwanza trial confirmed the already widely held belief that STI control almost surely slowed the spread of HIV. Recent meta-analyses of observational studies provide further corroboration [11,13,14]. The nine post-Mwanza trials do not confirm that hypothesis, but neither do they offer any basis for rejecting it, given the nature of statistical testing and, more importantly, given their multiple design flaws enumerated here. RCTs impose an unachievable standard of proof on the possible contribution of STIs to the spread of HIV.

The question that the trials were supposed to settle was a policy decision about allocating relatively abundant HIV funds for STI treatment, a critical but underfunded area of public health in sub-Saharan Africa. The issue was thus primarily political, not scientific. But public policy and science have different goals and different evidentiary standards [53]. Public policy is almost always forged in the absence of precise information [52,53]. There is no reason to require the same degree of certainty for policy decisions as for basic science or clinical research, particularly when known benefits and side effects favour implementation [52]. Neither science nor public health is served by the paralysis caused by the post-Mwanza trials. Observational studies provide adequate evidentiary basis for allocating some of the relatively abundant funds earmarked for HIV in sub-Saharan Africa to treat cofactor infections that promote HIV transmission.
